# Administration of Clinical COVID-19 Mouthwashing Protocol and Potential Modulation of Pediatric Oral Bacterial Prevalence of *Selenomonas noxia*: A Pilot Study

**DOI:** 10.3390/pediatric15030038

**Published:** 2023-07-11

**Authors:** Praneeti Sodhi, Yuxin Jiang, Summer Lin, Jackson Downey, Chase Sorenson, Melika Shayegh, Victoria Sullivan, Karl Kingsley, Katherine M. Howard

**Affiliations:** 1 Department of Advanced Education in Pediatric Dentistry, School of Dental Medicine, University of Nevada-Las Vegas, 1700 W. Charleston Boulevard, Las Vegas, NV 89106, USA; sodhip1@unlv.nevada.edu (P.S.); shayem1@unlv.nevada.edu (M.S.); victoria.sullivan@unlv.edu (V.S.); 2 Department of Clinical Sciences, School of Dental Medicine, University of Nevada-Las Vegas, 1700 W. Charleston Boulevard, Las Vegas, NV 89106, USA; jiangy18@unlv.nevada.edu (Y.J.); lins1@unlv.nevada.edu (S.L.); downej3@unlv.nevada.edu (J.D.); sorenc1@unlv.nevada.edu (C.S.); 3 Department of Biomedical Sciences, School of Dental Medicine, University of Nevada-Las Vegas, 1001 Shadow Lane Boulevard, Las Vegas, NV 89106, USA; katherine.howard@unlv.edu

**Keywords:** SARS-CoV-2, COVID-19 protocol, mouthwash, saliva, qPCR screening, *Selenomonas noxia*, dentistry, pediatric dentistry

## Abstract

Dental office protocols to combat the SARS-CoV-2 (COVID-19) pandemic include mouth washing for an extended 60 s, thereby reducing detectable oral virus. However, it is unclear whether this protocol has any effects on the newly identified periodontal pathogen and obesity-related bacterium often found among pediatric patients, *Selenomonas noxia*. To determine if the mouthwash protocol has any measurable effect on *S. noxia* amongst pediatric patients, clinical pediatric saliva samples were obtained from pediatric patients during routine visits for clinical care and treatment. Using an approved protocol, two saliva samples were collected on the same visit before and after chlorhexidine mouthwash (Sample A, Sample B). The third sample (Sample C) was taken at the recall appointment—usually between two and eight weeks later. A total of n = 97 pre-mouthwash samples, and an equal number of matching post-mouthwash samples (n = 97) were collected, with a small number of matching recall samples (n = 36) that were subsequently collected and identified. The demographic composition of the study sample was analyzed using Chi square statistics. Sample DNA from the matching pre-, post-, and recall collections (Sample A, Sample B, and Sample C) was isolated and screened using qPCR and validated primers, which revealed that 11.1% (n = 4/36) from Sample A tested positive for *S. noxia* with 0% (n = 0/36) of Sample B testing positive and 13.9% (n = 5/36) of the recall (Sample C) testing positive. In addition, comparative analysis of the qPCR cycle threshold data revealed relatively lower expression (quantity) of *S. noxia* DNA among the recall samples, as determined by two-tailed *t*-tests (*p*=0.004). These data and results provide new evidence for the oral prevalence of *S. noxia* among pediatric patients, while also demonstrating that the COVID-19 protocol of mouth washing prior to clinical treatment for periods extending up to 60 s may be sufficient to reduce the levels of detectable *S. noxia*—at least temporarily. More research will be needed to determine whether these effects may be limited to the short- or may exhibit more lasting effects in the long-term.

## 1. Introduction

Many biosafety protocols were introduced during the SARS-CoV-2 pandemic to improve patient safety and limit the transmission of the virus among patients and healthcare personnel [1,2]. The most common practices to combat cross contamination and infection included the use of face shields and respiratory N95 protective face masks for health care workers, changes to the procedures and protocols that generate significant aerosols, as well as the introduction of an extended clinical mouthwash protocol for use by patients (consisting of gargling or rinsing for a minimum of thirty seconds but lasting up to sixty seconds) to reduce the amount of detectable SARS-CoV-2 virus in oral secretions [3,4]. The use of this specific pre-procedural dental office protocol to combat the SARS-CoV-2 (COVID-19) pandemic, by mouth washing for a prolonged time period of up to 60 s or more, has been found to demonstrate clinical efficacy in reducing the amount of detectable viral levels in salivary secretions and oral samples for up to one hour following completion [5,6].

More specifically, some of these studies have evaluated not only the effectiveness of this protocol, but also of the many mouth washing and rinsing agents, with active ingredients that include povidone-iodine, hydrogen peroxide, and chlorhexidine among others to significantly reduce SARS-CoV-2 levels in saliva [7,8]. Most of the studies evaluated have demonstrated dramatic and effective reductions in detectable virus levels in saliva and other oral secretions immediately after the pre-procedural mouth washing protocol, regardless of which of these active ingredients were included in the commercial product tested [9,10,11]. These studies and their supporting data confirm previous observations regarding the anti-viral capacity of many mouthwash and rinsing antiseptics against other enveloped viruses including herpes simplex virus (HSV) and the respiratory pathogen adenovirus [12,13,14].

Although these procedures were performed to reduce the risk of SARS-CoV-2 transmission, there have been studies that have examined the potential for extended or prolonged mouth washing protocols to reduce the microbial burden of other oral pathogens, including oral bacteria [15,16]. In fact, two more recent studies have confirmed that pre-procedural mouthwashes, such as those employed to combat the COVID-19 pandemic, are effective at reducing both detectable oral viruses—as well as other types of oral microbes including oral bacteria, such as important Gram-positive *Streptococcus* species. (*S. mutans*, *S. salivarius*, and *S. sanguis*) and *Lactobacillus* (*L. casei*) [17,18]. However, few studies to date have evaluated the specific effects of an extended mouth washing protocol on other important oral microbes including several of the more clinically relevant Gram-negative species [16,17].

For example, it is unclear whether this protocol has any effects on the newly identified periodontal pathogen and obesity-related bacterium, *Selenomonas noxia* (*S. noxia*) [19,20]. This Gram-negative anaerobic organism is strongly associated with subgingival disorders including periodontitis and gingivitis, as well as root surface caries [21,22,23]. Moreover, recent evidence has also suggested that this organism may also be present in supragingival oral biofilms, as well as on the dorsum of the tongue that may contribute to the seeding of the gastrointestinal tract and may contribute both directly and indirectly to obesity-specific metabolic disorders among these patients [24,25]. As more and more pediatric patients are diagnosed as clinically overweight and obese, and growing evidence demonstrates that species that keystone including *S. noxia* may be important modulators of these phenotypes, understanding how and when changes in clinical protocols alter the prevalence of this organism become increasingly more important to oral health researchers and clinical research professionals [24,25].

The recent discovery by this group of novel oral biofilm sites for this organism in supragingival biofilm and on the dorsum of tongue, where they may be exposed to the mechanical forces of gargling and rinsing as well as the active ingredients contained within these products could suggest that this organism might be more susceptible to the extended mouth washing protocol instituted for the COVID-19 pandemic than would be expected given their traditional locations within the gingival crevice and periodontal pockets. However, no studies to date have evaluated this possibility specifically evaluating any changes in the presence (or absence) of this organism. Based upon this information, the primary objective of this project was to determine if the standard COVID-19 mouthwash protocol has any measurable effect on *S. noxia* amongst pediatric patients to provide evidence to support or refute the null hypothesis that no measurable effect would be observed. This information may be particularly important for pediatric researchers and clinicians that are evaluating the clinical protocols and procedures that are most effective at reducing risk and increasing oral health outcomes among pediatric patients specifically.

## 2. Materials and Methods

### 2.1. Study Approval

This project was conducted in accordance with the Declaration of Helsinki with the review and approval by the Institutional Review Board (IRB) at the University of Nevada, Las Vegas (UNLV) under protocol 1717625-1 titled “Retrospective analysis of microbial prevalence from DNA isolated from saliva samples originally obtained from the University of Nevada, Las Vegas (UNLV) School of Dental Medicine (SDM) pediatric and clinical population” on 3 March 2021. The original protocol for the collection of saliva samples was reviewed and approved by the UNLV IRB under OPRS#1305-4466M protocol “The Prevalence of Oral Microbes in Saliva from the UNLV School of Dental Medicine Pediatric and Adult Clinical Population”.

### 2.2. Human Subjects and Informed Consent

In the original saliva collection protocol, patient samples were collected from voluntary participants in the UNLV-SDM clinic. Inclusion criteria included adult patients who voluntarily chose to participate and provided Informed Consent. Inclusion criteria for pediatric patients (over seven years of age) included those patients who voluntarily chose to participate and provided Pediatric Assent, as well as Informed Consent from the appropriate consenting guardian or parent. Exclusion criteria included any patients (adult or pediatric) who declined to participate and any person not in treatment at the UNLV-SDM clinic. In brief, unstimulated saliva was collected in sterile collection tubes. Prior to the clinical sample collection, each tube was labeled with a randomly generated, non-duplicated number in order to prevent any patient-specific identifying information from being collected with any patient samples. Basic demographic information including patient age, race or ethnicity, and sex were noted for each clinical collection before transferring any collected samples to a biomedical laboratory for long-term storage and further processing.

### 2.3. DNA Isolation

To facilitate the molecular screening, saliva samples were first thawed and processed to isolate the DNA using the TRIzol reagent from Invitrogen (Waltham, MA, USA), as previously described in [26,27]. In brief, samples were thawed at room temperature and then vortexed. A standardized amount of 500 µL from each sample was aliquoted into a sterile microcentrifuge tube and mixed with an equal volume of 500 µL of TRIzol reagent. To this mixture, 200 µL of molecular biology grade Chloroform from Fisher Scientific (Waltham, MA, USA) was added and incubated on ice for ten minutes prior to centrifugation at 12,000× *g* relative centrifugal force (RCF) at 4 °C in a refrigerated microcentrifuge from Eppendorf—Model 5425 (Hamburg, Germany). Following centrifugation and separation, the upper aqueous phase (approximately 300 to 400 µL) was transferred to a new sterile microcentrifuge tube and an equal volume of molecular biology grade Isopropanol from Fisher Scientific (Waltham, MA, USA) was added to precipitate the DNA from each sample [26,27]. All samples were then centrifuged again for ten minutes at 12,000× *g* RCF at 4 °C in a refrigerated microcentrifuge from Eppendorf—Model 5425 (Hamburg, Germany). Following this centrifugation procedure, the isopropanol was removed prior to washing the remaining DNA pellet with 100% ethanol. Each DNA sample was then centrifuged for an additional five minutes at 12,000× *g* RCF at 4 °C in a refrigerated microcentrifuge from Eppendorf—Model 5425 (Hamburg, Germany) and ethanol was removed from the pellet prior to resuspension with nuclease-free water obtained from Fisher Scientific (Waltham, MA, USA). The DNA concentration and purity were both determined using a NanoDrop 2000 spectrophotometer from ThermoFisher Scientific (Waltham, MA, USA) using absorbance readings at A260 nm and A280 nm.

### 2.4. qPCR Screening

Each of the processed DNA samples was then screened for the presence of *S. noxia* using quantitative polymerase chain reaction (qPCR) and the QuantStudio system from Applied Biosciences (Waltham, MA, USA). In brief, sample DNA was screened using the Fast SYBR green master mix from Fisher Scientific (Waltham, MA, USA) involving 12.5 µL of 2X SYBR green master mix, 6.0 µL of nuclease-free water from Fisher Scientific (Waltham, MA, USA), 1.75 µL of forward and reverse primer diluted to a standard concentration of 10 µM, and 2.0 µL of sample DNA. Cycle settings were derived from the manufacturer protocol, involving a 20 s activation set at 95 °C followed by 40 cycles of denaturation at 95 °C for five seconds and extension at 60 °C for thirty seconds, as previously described [25,26,27]. Validated primers synthesized by Eurofins Scientific (Louisville, KY, USA) included (Table 1):

### 2.5. Statistical Analysis

Basic descriptive statistics were compiled for the study sample. Analysis of demographic data from the study sample was compared with overall demographic data from the clinic using Chi square analysis, which is appropriate for categorical, non-parametric analysis. Screening results (*S. noxia*-positive, *S. noxia*-negative) were also analyzed using Chi square analysis using the online statistical software package Chi Square Calculation, Version 9 from GraphPad (San Diego, CA, USA). All significance values were set at alpha = 0.05.

Finally, for the purpose of calculating the sample size minimum that would be appropriate for this type of salivary-based qPCR microbial screening, the DNA extraction recovery rate (sample-limiting step) of 90–95% was used to determine the maximum expected experimental difference of 10% or 0.10. Using this information with a Power (p) of 0.90 and a significance level of alpha = 0.95, the minimum total sample size of N = 50 was calculated for this study [26,27].

## 3. Results

A total of n = 97 clinical samples of individual patients were identified for this study (Table 2). The demographic analysis of the study sample found nearly equal percentages of females and males, which was similar to the overall distribution of males and females found in the main patient clinic, *p* = 0.5422. In addition, the proportion of minority patients in the study sample (77.3%) closely matched the overall proportion of minority patients identified in the overall clinic population (75.3%), *p* = 0.6379. The majority of non-White patients self-identified as Hispanic or latino (58.8%), which also closely matched the overall percentage of Hispanics from the clinic population (52.4%). Finally, the average age of the study sample patients identified was 9.17 years (range 5 to 16 years), which was similar to the average age of the overall pediatric clinic population of 9.04 years.

DNA was isolated from each of the clinical Sample A, Sample B, and Sample C (Pre-mouthwash, Post-mouthwash, and Recall appointment) samples, n = 36 (Table 3). Analysis and comparison of the DNA concentration revealed Sample A (Pre-mouthwash) had an average of 1141.74 ng/µL, which was higher than the average observed among Sample B (Post-mouthwash) of 883.94 ng/µL (*p* = 0.0039) but lower than the average concentration found among Sample C (Recall) of 1350.9 ng/µL (*p* = 0.0359). DNA purity as measured by the absorbance ratio of A260 nm and A280 averaged between 1.72 and 1.75 between Sample A, Sample B, and Sample C, which met the minimum requirements for molecular screening using qPCR.

**Figure 1:**
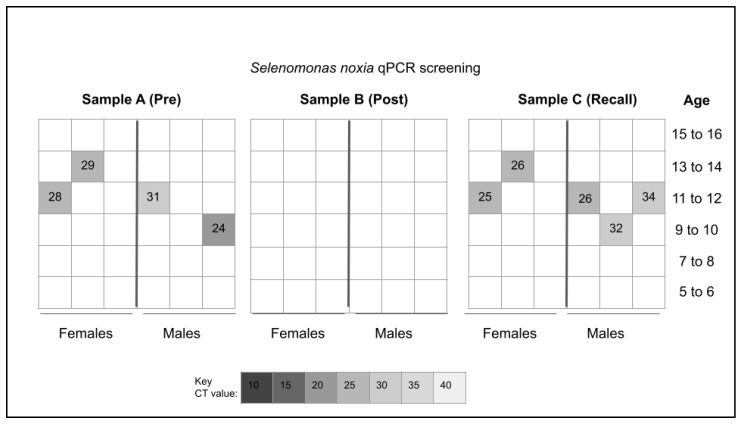
qPCR heat map for *Selenomonas noxia* (SN) sample screening. Analysis revealed n = 4/36 or 11.1% of pre-mouthwash analysis (Sample A) harbored DNA specific for *S. noxia* (SN) with none of the post-mouthwash analysis (Sample B) testing positive n = 0/36. Analysis of the recall or follow up (Sample C) revealed n = 5/36 or 13.9% tested positive for *S. noxia* (SN) including three of the original four *S. noxia* (SN)-positives from Sample A—as well as two additional previously *S. noxia* (SN)-negative samples.

Samples from each of the saliva collection groups (Pre-mouthwash, Post-mouthwash, and Recall) were screened for the presence of the Gram-negative bacterium *S. noxia* (SN) using validated qPCR primers (Figure 1). This analysis revealed that n = 4/36 or 11.1% of pre-mouthwash samples harbored *S. noxia* (SN), which was equally distributed among males and females in this sample. None of the *S. noxia* (SN)-positive samples were found among the youngest age group (5 to 6, 7 to 8 years) or among the oldest age group (15 to 16 years). Analysis of the post-mouthwash samples (following the completion of the 30 to 60 s SARS-CoV-2/COVID-19 mouthwash protocol) revealed that none of the samples harbored *S. noxia* (SN) above the limit of detection. However, screening of the recall samples from the follow up appointments revealed that n = 5/36 or 13.9% harbored this organism including three of the original four samples and two newly *S. noxia* (SN)-positive samples.

**Figure 2:**
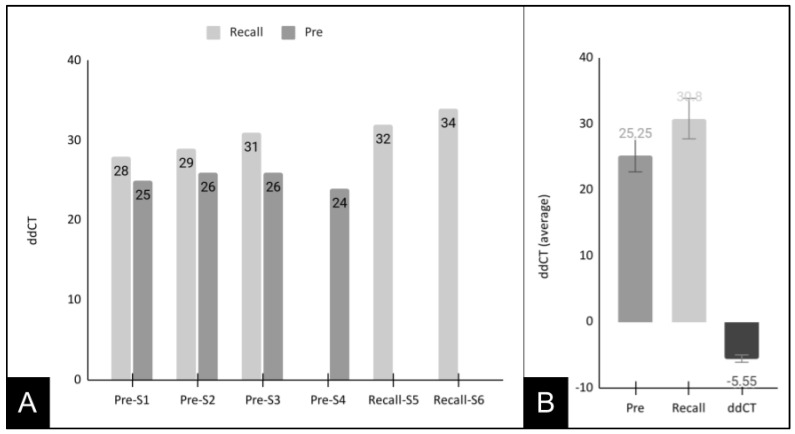
Analysis of cycle threshold (CT) values for *S. noxia*-positive samples. (**A**) Sample comparisons demonstrated pre-mouthwash *S. noxia*-positive samples (CT matched = 25, 26, 26, and CT unmatched = 24—average 25.25) were lower than recall samples (CT matched = 28, 29, 31, and CT unmatched 32, 34—average 30.8). (**B**) Average CT for the pre-mouthwash samples (25.25) was much lower than among the recall samples (30.8), with a significant difference of CT = −5.55, *p* = 0.004.

To more closely evaluate these results, the *S. noxia* (SN)-positive sample data from the pre-mouthwash and recall samples were sorted and graphed for direct comparison (Figure 2). These data demonstrated that the cycle threshold values for the pre-mouthwash *S. noxia* (SN)-positive samples (CT = 25, 26, 26, 24—average 25.25) were much lower than the three-matched and two unmatched recall samples (CT = 28, 29, 31, 32, 34—average 30.8). The average difference in CT values between the pre-mouthwash and recall samples was 5.55, which was statistically significant, *p* = 0.004.

## 4. Discussion

The discovery of novel oral biofilm sites for *S. noxia* outside of the gingival crevice and periodontal pockets has opened the possibility that this organism might be susceptible, at least in part, to the extended mouth washing protocol administered to combat SARS-CoV-2 (COVID-19). The primary objective of this project was to determine if the mouthwash protocol and this extended period of rinsing and gargling had any measurable effect on *S. noxia* amongst pediatric patients. This study revealed significant and immediate changes in the detectable oral prevalence of this organism that was comparable to other previously conducted studies that evaluated the presence of Gram-negative bacteria and also included extended mouth washing or rinsing protocols [28,29]. As other studies have attempted to determine the effectiveness of oral hygiene instructions and protocols designed to limit or eliminate other species of pathogenic Gram-negative bacteria in specific subsets of patients with specific clinical parameters, the strict adherence to the COVID-19 mouth washing protocol offered a unique opportunity to evaluate these potential effects in a much wider variety of pediatric patients with differing oral microbial constituents [30,31]. The findings that the presence of oral *S. noxia* may be temporarily reduced or eliminated among pediatric patients using this protocol and that the comparative levels of *S. noxia* among recall samples may be lower than detected in the original sample collection are significant and important findings that could have significant implications for clinical care and protocol recommendations within this patient population.

However, despite the significance of these results, some issues were identified with regard to the prevalence of this organism among these pediatric patients over the course of these observations. First, the immediate and dramatic reductions observed immediately after the mouth washing protocol do not appear to be sustained over time, as most of the patients who initially tested *S. noxia*-positive before the protocol were found to harbor this organism once again at the recall appointment. This may suggest that the SARS-CoV-2/COVID-19 protocol may only induce a temporary effect on *S. noxia* that may be more specifically related to the extension of mouth washing time, an effect which has been demonstrated in other studies to dramatically reduce the prevalence of other pathogenic supra-gingival oral bacteria if practiced regularly [32,33,34].

Another issue identified was that more patients exhibited *S. noxia*-positive results at the recall appointment than was observed in the initial pre-mouthwash screening, which may be due to natural variation in oral microbial prevalence over time [35,36,37]. However, these results may also suggest that the removal of commensal and other bacterial constituents through the extended mouth washing protocol may provide opportunities for *S. noxia* to colonize biofilms and other oral sites following this procedure [38,39]. Due to the lag time and significant variation between the initial appointment and saliva collections and the follow up appointments, it may be impossible to determine from the existing data obtained from this study which of these mechanisms may be most likely to explain these observations [40,41,42].

These concepts introduce variables that may be considered intrinsic limiting factors to the inferences that can be drawn from these observations. For example, this study was an observational retrospective analysis of saliva samples collected using the strict COVID-19 mouth washing protocol and did not use an experimental model to evaluate the potential effects of different active agents within the mouth washing agents such as chlorhexidine, povidone-iodine, and fluoride [43,44]. The incorporation of a prospective, experimental model in future studies evaluating these potential effects would help to determine what variables (including extended time of gargling or rinsing, frequency of protocol administration including daily or multiple times per day, or constituent active ingredients—or some combination thereof) may be the most effective at reducing or eliminating this organism from pediatric patients. In addition, some recently introduced compounds have also been demonstrated to have significant influences on the oral environment, including probiotics that have demonstrated the ability to modify clinical and microbiological parameters among some periodontal patients, which may also be considered useful variables to include in future prospective trials to evaluate potential effects on *S. noxia* [45,46,47].

Another potential limitation was that this study did not evaluate the potential effects of prolonged exposure to repeated daily periods of mouth rinse over longer and more standardized periods of time, which may have the potential to exhibit more robust and sustained effects on *S. noxia* as has been observed in other previous studies and trials of Gram-positive and Gram-negative oral bacteria using mouthwash and mouth rinsing agents [28,48,49]. Finally, this study was not able to control for oral hygiene and oral health status among this low-income, pediatric population—which may have had the potential to influence these observations as barriers and challenges to promote oral health have been observed within this specific clinical patient population [50,51,52].

These findings are particularly important as the presence of this organism is not only associated with oral disorders but is also strongly associated with long-term negative health outcomes including obesity [53]. In fact, previous studies have revealed that nearly 99% of obese patients could be identified merely by the presence of this oral bacterium when it exceeds greater than 1.5% of total oral bacterial species [24,25,53]. Because *S. noxia* has been demonstrated to be capable of metabolizing and fermenting “indigestible” carbohydrates, thereby extracting more calories from fiber-containing and low-calorie foods, an understanding of the prevalence of this organism might provide useful clinical data regarding both oral and systemic disease risks among pediatric dental patients [54]. Finally, as more studies confirm the presence of *S. noxia* among pediatric patient populations a more complete and comprehensive view of the parameters and risk factors that influence the distribution of this organism may be revealed—an important step for improving oral health and systemic health outcomes for pediatric patients.

## 5. Conclusions

This study provides evidence that the SARS-CoV-2 mouth washing protocol may be capable of modulating other important species of bacteria such as *Selenomonas noxia* (at least temporarily), in addition to reducing detectable oral COVID-19 [55,56]. Although the number of clinical samples evaluated in this initial pilot study was not sufficient to make broad inferences, these results are sufficient to confirm that additional prospective and experimental studies are needed to determine whether these effects are limited or temporary or if they have more long-term effects such as shifting microbial prevalence and *S. noxia* more specifically among pediatric patients over time.

## Figures and Tables

**Table 1:** Validated primer sequences.

Positive Control, Bacterial 16S rRNA	
Forward 16S rRNA primer	5′-ACG CGT CGA CAG AGT TTG ATC CTG GCT-3′
Reverse 16S rRNA primer	5′-GGG ACT ACC AGG GTA TCT AAT-3′
Selenomonas noxia (SN) primer	
Forward primer SN-F1	5′-TCT GGG CTA CAC ACGT ACT ACA ATG-3′
Reverse primer SN-R1	5′-GCC TGC AAT CCG AAC TGA GA-3′

**Table 2:** Demographic analysis of study samples.

Demographic Characteristic	Study Sample (n = 97)	UNLV—SDM Clinic Summary	Statistical Analysis
*Sex*			
Female	49.5% (n = 47/97)	52.8%	X^2^ = 0.371, d.f. = 1
Male	50.5% (n = 50/97)	47.2%	*p* = 0.5422
*Race/Ethnicity*			
non-Minority (White)	22.7% (n = 22/97)	24.7%	X^2^ = 0.221, d.f. = 1
Minority (non-White)	77.3% (n = 75/97)	75.3%	*p* = 0.6379
Hispanic/Latino	58.8% (n = 57/97)	52.4%	
Black/African American	11.3% (n = 11/97)	12.2%	
Asian/Pacific Islander	3.1% (n = 3/97)	3.8%	
Native American/American Indian	4.1% (n = 4/97)	0.1%	
Age			
Average Age Range of Age	9.17 years (5 to 16 years)	9.04 years (1 to 18 years)	

**Table 3:** DNA analysis of saliva samples.

Study Sample	DNA Concentration (Average and Standard Deviation)	DNA Purity A260:A280 Ratio
Sample A (Pre-mouthwash) T1, n = 36 Initial appointment	Average: 1141.7 ng/µL STD +/− 38.5 ng/µL	Average: 1.72 Range: 1.65–1.85
Sample B (Post-mouthwash) T2, n = 36 Initial appointment	Average: 883.9 ng/µL STD +/− 41.7 ng/µL Two-tailed *t*-test T1:T2, *p* = 0.0039	Average: 1.74 Range: 1.61–1.87
Sample C (Recall) T3, n = 36 Recall appointment	Average: 1350.9 ng/µL STD +/− 41.7 ng/µL Two-tailed *t*-test T1:T3, *p* = 0.0359	Average: 1.75 Range: 1.60–1.84

## Data Availability

The data presented in this study are available on request from the corresponding author.
